# Is there a role for immune checkpoint blockade in metastatic uveal melanoma?

**DOI:** 10.1186/1479-5876-13-S1-K20

**Published:** 2015-01-15

**Authors:** Bastian Schilling, Tim Schneider, Inga Möller, Antje Sucker, Annette Paschen, Dirk Schadendorf, Klaus G Griewank

**Affiliations:** 1 Department of Dermatology, University Hospital, West German Cancer Center, University Duisburg-Essen, Essen, Germany; 2 German Cancer Consortium (DKTK), Germany

## Introduction

Uveal melanoma is the most common intraocular malignancy in adults with a disease specific mortality rate of ~40%. Oncogenic mutations in *GNAQ* and *GNA11* were recently identified as driver mutations in ~90% of uveal melanoma. While localized disease can be effectively treated by surgery or radiotherapy, treatment options for metastatic uveal melanoma are limited. To investigate the interplay between uveal melanoma and the hosts´ immune system and to test immunotherapeutic approaches, we have established an syngenic mouse model of *GNAQ* oncogene-driven melanoma.

## Materials and methods

Melan-a cells were transduced with retroviruses expressing an activating Q209L mutation of *GNAQ*. Tumor formation was measured with calipers after subcutaneous inoculation of the cell line in C57BL/6 or RAG2^-/-^ mice. Splenocytes were harvested at the time of sacrifice. For NK cells depletion, an anti-NK1.1 antibody was administered weekly. Murine anti-CTLA-4 antibodies (100µg), anti-PD-1 antibodies (250µg) or the combination of both were given weekly in a therapeutic setting. Peripheral blood mononuclear cells (PBMC) from uveal melanoma patients were obtained after informed consent and analyzed by flow cytometry.

## Results

Melan-a cells with mutant but not with wt *GNAQ* form tumors when injected into mice, resulting in a model of G-protein-driven experimental murine melanoma (GEM). No significant difference in tumor growth was observed between C57BL/6 or RAG2^-/-^ mouse strains. Elimination of NK cells in RAG2^-/-^ mice led to increased tumor growth compared to non-depleted controls (p<0.05). Multi-color flow cytometry revealed a significant increase of CD11b^+^Gr-1^int^ myeloid-derived suppressor cells (MDSC) as well as regulatory T cells (Treg) in tumor-bearing animals as compared to non-tumor-bearing animals. In PBMC from patients with metastatic uveal melanoma, an increased frequency of monocytic MDSC and Treg was found as compared to healthy controls. In the GEM model, concurrent administration of an anti-CTLA-4 antibody and an anti-PD-1-antibody does delay tumor growth significantly (p<0.05) while monotherapy with either agent did not. No reduction in the accumulation of Treg and MDSC was found when comparing treated and untreated mice.

## Conclusion

In the syngenic GEM model, tumor growth seems to be controlled by NK cells and can be delayed by dual immune checkpoint blockage. In this model, tumor formation is accompanied by an immunosuppressive leukocyte network also found in patients with metastatic uveal melanoma. Specific elimination of these cells might therefore synergize with immune checkpoint blocking antibodies. This hypothesis needs to be tested in future studies to better define its treatment potential for patients with advanced uveal melanoma.

